# Manganese Volatilization and Its Influence on Low-Pressure Carburizing Process Effects

**DOI:** 10.3390/ma18225173

**Published:** 2025-11-14

**Authors:** Radomir Atraszkiewicz, Piotr Zgórniak

**Affiliations:** 1 Institute of Materials Science and Engineering, Lodz University of Technology, Stefanowskiego 1/15, 90-537 Lodz, Poland; 2 Institute of Machine Tools and Production Engineering, Lodz University of Technology, Stefanowskiego 1/15, 90-537 Lodz, Poland; piotr.zgorniak@p.lodz.pl

**Keywords:** volatilization, manganese, heat treatment, carburizing, deformation

## Abstract

The presented research quantitatively characterizes the depth and fraction of Mn volatilization under LPC conditions for the first time and its influence on surface layer properties. It has been proven that this phenomenon increases with holding time at carburizing temperature and that the intensity of this phenomenon can be limited by using step carburizing, where carburizing stages applied during heating to the target temperature shorten the exposure time at the target temperature. Simultaneously, an increase in carbon concentration in the surface layer causes a limitation of Mn escape due to attractive interactions with carbon atoms. All the above aspects cause the kinetics of the carburizing process, and consequently the effect of heat treatment after carburizing, to depend on changes in the chemical composition of the surface layer. It has also been proven that the influence of manganese volatilization decreases with increasing carburized layer thickness, regardless of the process implementation method.

## 1. Introduction

Low-pressure carburizing, as an alternative process to traditional gas carburizing, is a process that brings many benefits. Due to higher process temperatures and a reduction in internal oxidation problems, such a process represents significant progress in the field of heat treatment [1,2,3]. The use of acetylene and/or ethylene in the carburizing mixture with hydrogen [4] contributes to the formation of uniform carburized layers, even in deep blind holes, thanks to the high decomposition energy of acetylene [5]. However, on the other hand, the process is a non-equilibrium process, precisely due to the nature of carbon carrier decomposition and a much higher carbon flux, which is, on average, five times higher than in gas carburizing with potential control [6,7]. Hence, the implementation of low-pressure carburizing typically involves the application of alternating boost stages, in which an atmosphere containing carbon carriers is introduced into the chamber, and diffusion stages, in which the absorbed carbon diffuses into the material. In this way, the carbon concentration on the surface is controlled during the process, as well as the dissolution of carbide structures formed on the surface [8,9].

For the low-pressure carburizing process, the chemical composition of the treated material is of great importance. The influence of alloying elements on the carbon diffusion process is well known, where individual elements can either accelerate it (Ni, Si) or limit its intensity (Cr, Mo, Ti) [10,11,12,13]. These elements also influence the nature and kinetics of the heat treatment process applied after carburizing to obtain final mechanical and strength properties. It is also worth considering the fact that in low-pressure carburizing, a phenomenon of chemical composition change during the process appears, which is not extensively commented on or studied in the literature [14]. It is called volatilization and results from pressure reduction at high temperature. It is commonly known that under such conditions, various alloying elements can already evaporate due to high vapor pressure [15]. Manganese plays a particular role here, for which, under the thermodynamic parameters presented in this publication, the phenomenon of escape from the material to the atmosphere and effusion in the material already occurs [16,17]. The phenomenon of manganese volatilization is widely described in research related to the metallurgy of alloys, where its influence on the subsequent mechanical properties of the alloy is described [18,19,20,21]. In the case of the solid-state analysis, no studies were found regarding the variation in Mn concentration in the surface layer. Only the influence of different concentrations on properties was described in the literature, which indicated significant interactions of Mn with carbon and oxygen [22,23]. It was also proven that Mn affects the kinetics of the solid-state diffusion process, and it is an element that inhibits diffusion processes with increasing concentration [13,24]. However, these studies do not concern the carburizing process and the influence of chemical composition changes in the surface layer on the process itself.

This publication demonstrates that carbon pre-saturation effectively suppresses Mn volatilization and establishes a mechanistic pathway linking Mn volatilization → compositional gradient → CCT evolution → mechanical response → macroscopic deformation.

## 2. Materials and Methods

### 2.1. Materials

**Figure 1:**
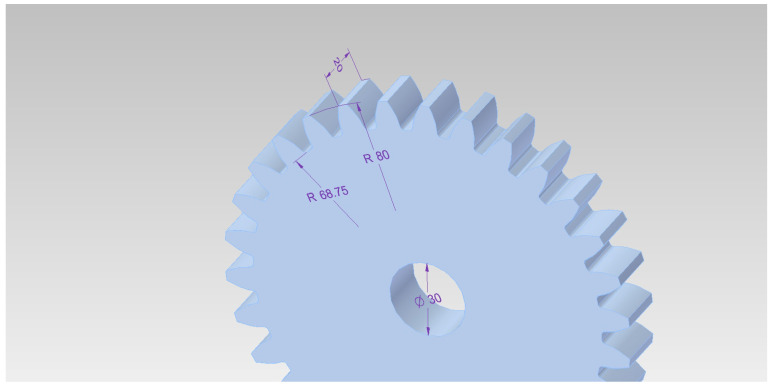
Geometry of gears used for testing (dimensions in mm).

The samples used for testing were gears with module 5, made of EN16MnCr5 material. Their geometry and main dimensions are presented in Figure 1.

The chemical composition of the material used is presented in Table 1.

The gears prepared for testing were manufactured by machining (gear shaping), and then normalizing annealing was carried out to remove stress. Subsequently, four low-pressure carburizing processes [25], were carried out: two with preliminary carburizing for one minute at temperatures of 850, 900, and 950 °C, after which target carburizing was carried out at a temperature of 1000 °C (these processes were called step carburizing—SC), and two carried out at a constant temperature of 1000 °C, designated as CTC-constant temperature carburizing [26], with one process in each group carried out for an effective layer thickness of 0.7 mm and the second for a thickness of 1.0 mm. In both cases, the carburized layer criterion was defined as 0.4 wt.%C. A carbon concentration on the surface equal to 0.8 wt.%C was assumed. The carburizing and holding times at given temperatures for individual processes are presented in Table 2.

A universal vacuum furnace of type VPT-4022/24IQN (Seco/Warwick Group, Swiebodzin, Poland) was used to carry out the processes. The pressure of the carburizing atmosphere in boost segments was 300–1000 Pa, and in diffusion segments it was 50 Pa. After carburizing, the parts were subcooled to the quenching temperature appropriate for the material (860 °C) and then cooled in a nitrogen stream under a pressure of 0.9 MPa. In the final stage, stress-relief tempering at a temperature of 190 °C was applied.

### 2.2. Glow Discharge Optical Emission Spectroscopy Analysis

The chemical composition of the material was investigated using the glow discharge optical emission spectroscopy method, using a LECO GDS-850A (LECO Corporation, St. Joseph, MI, USA) device.

The measurement parameters for the measured materials in the case of the above-mentioned methods were voltage Ug = 700 V, intensity Ig = 30 mA, and pressure pg = 1.3 kPa. Measurements were made with a frequency of 50 Hz. The measurement accuracy of the method used is at the level of ±0.011%. The QDP (quantitative depth profiling) technique was used in research for alloying elements, and the bulk method for determining changes in carbon concentration.

### 2.3. Microstructure and Hardness Assessment

Hardness tests were carried out using a KB Prüftechnik GmbH FA (Hochdorf-Assenheim, Germany) hardness tester, type KB105VZ-FA, and KB Prüftechnik Hardwin XL v2.4.15 software. Measurements were made at a load of 0.05 kG.

### 2.4. Residual Stress and Retained Austenite Content Measurements by XRD

Residual stress measurements in the specimens were performed through the sin^2^ψ X-ray technique in ω geometry, utilizing a PROTO iXRD device (Proto Manufacturing Inc., Taylor, MI, USA) fitted with dual position-sensitive semiconductor detectors. Characteristic X-radiation with a wavelength of λ = 2.29 Å was generated from a Cr anode tube serving as the X-radiation source. The analysis focused on tracking the positional shift in the (211) iron characteristic peak, located at 2θ = 156.4°. For computational purposes, X-ray elastic constants of ½S_2_ = 5.92 1/TPa and S_1_ = −1.27 1/TPa were employed. The measurement was conducted in a region defined by a φ = 2 mm diameter collimator.

For XRD-based characterization of retained austenite concentration, the identical diffractometer described previously was employed. The instrument collected four diffraction peaks: two corresponding to the ferrite/martensite phase—(200) and (211) at 106° and 156° 2θ, respectively—and two for the austenite phase at 79° (200) and 128° (220). The volume percentage of retained austenite present in the specimen was determined by comparing the intensities of these four peaks. All computational procedures and methodologies followed the ASTM E-975 [27] standard.

### 2.5. Geometry Changes Assessment

The gear components were evaluated for the following key geometric characteristics:

- Thickness of teeth;
- Bore diameter distortion;
- Outer radial run-out at the pitch diameter.

A Carl Zeiss Jena (Jena, Germany) gear measurement system was utilized to conduct these measurements.

Bore diameter distortion, radial run-out, and tooth thickness were assessed using an electronic gauge with a scale resolution of 0.001 mm. The overall measurement uncertainty was 0.003 mm.

## 3. Results and Discussion

### 3.1. Chemical Composition

**Figure 2:**
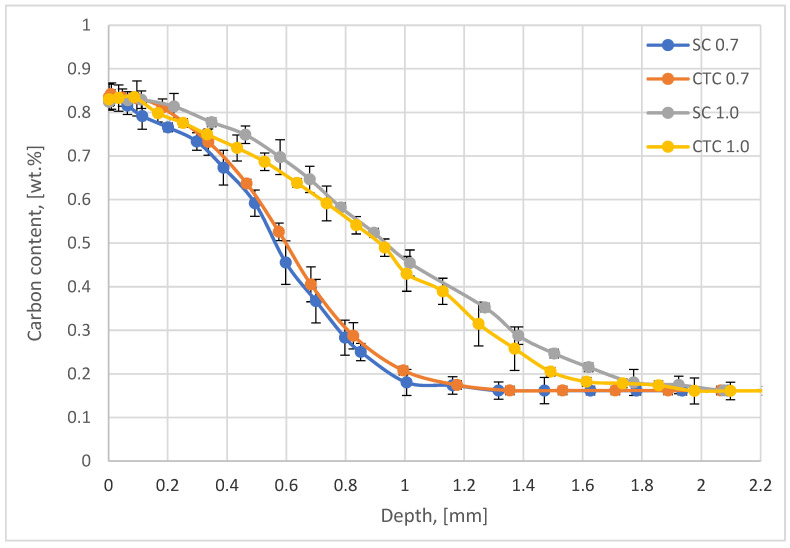
Carbon profiles for step (SC) and constant temperature (CTC) low-pressure carburizing.

First, studies of carbon profiles for step carburizing and constant temperature carburizing were carried out. Figure 2 presents the results of tests using the optical emission spectrometry with the glow discharge method using the bulk option.

In the case of processes performed using step carburizing (SC) as well as traditional constant temperature (CTC), the effective thickness of the carburized layer for the 0.4 wt.% criterion is 0.7 mm, which is identical to the assumptions. In the case of processes carried out for the assumed depth of 1.0 mm, in both analyzed variants, an effective layer thickness at the level of 1.1 mm was obtained for the criterion of 0.45 wt.%C. In all four cases considered, the carbon concentration on the surface is approximately 0.83 wt.%C, with an assumed criterion of 0.8 wt.%C.

**Figure 3:**
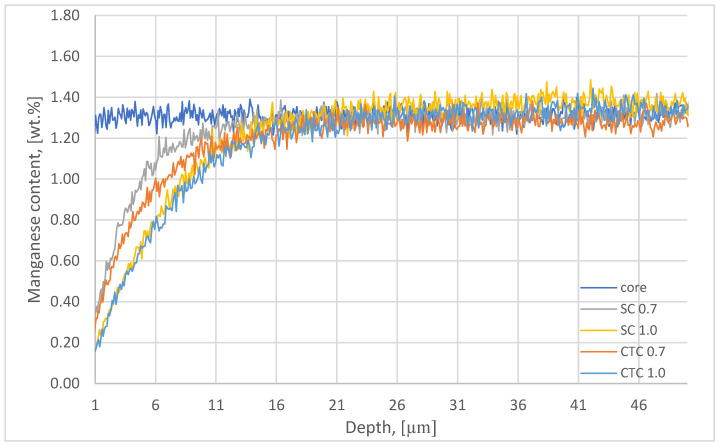
Manganese content plot on the surfaces and in the cores of wheels subjected to carburizing.

In the next stage, the chemical composition studies of the surface layer were supplemented with GDOES analysis of alloying elements such as Mn, Cr, and Si; however, in this case, the QDP technique described in Section 2 was used. The results for manganese are presented in Figure 3.

The reduction in partial pressure in diffusion segments caused the manganese to escape from the near-surface layer [21,28,29]. This effect results from the high vapor pressure of Mn under conditions of elevated temperature and reduced pressure [15]. In the case of the step carburizing process to a depth of 0.7 mm, this effect is visible to a depth of approximately 17 µm. For the SC process, in the case of a process designed for an effective layer thickness of 1 mm, changes in Mn concentration in the surface layer reach 40–50 µm. It should be noted that the total time of diffusion segments at 1000 °C is 40 min for the SC0.7 process, and for the SC1.0 process, it is 137 min. For the constant temperature carburizing process to a depth of 0.7 mm, changes in Mn concentration in the surface layer reach 25 µm, and for the CTC1.0 process, changes in manganese concentration are similar to those represented by the SC1.0 process, where the influence of manganese escape to a depth of approximately 40–50 µm from the surface was observed. For these processes, the time of diffusion segments was 55 min for CTC0.7 and 161 min for the CTC1.0 process, respectively. The influence of the carburizing method on the intensity of manganese escape can be clearly observed for the SC0.7 and CTC0.7 processes, where, for example, at a distance of 5 µm from the surface, the Mn concentration is 1.02 wt.% and 0.86 wt.%, respectively. This corresponds to a change in Mn concentration, relative to the initial composition, of 22% and 37%. In the case of processes carried out for a layer thickness of 1 mm, in both cases, regardless of the process implementation method, it can be assumed that changes in Mn concentration in the surface layer are comparable and for a depth of, for example, 5 µm from the surface amount to 50% of the initial value (Mn concentration is ~0.67 wt.%). The manganese concentration on the surface for processes carried out for a layer thickness of 0.7 mm is ~0.23 wt.%, which corresponds to a concentration change relative to the alloy before treatment of approximately 83%. For processes carried out for a layer thickness of 1 mm, the Mn concentration on the surface is ~0.16 wt.%, which is a change of 89%.

The influence of treatment time is therefore clearly noticeable, while the influence of the process implementation method is clearly noticeable for smaller values of effective carburized layer thickness and disappears for layers designed with larger values of effective carburized layer thickness.

**Figure 4:**
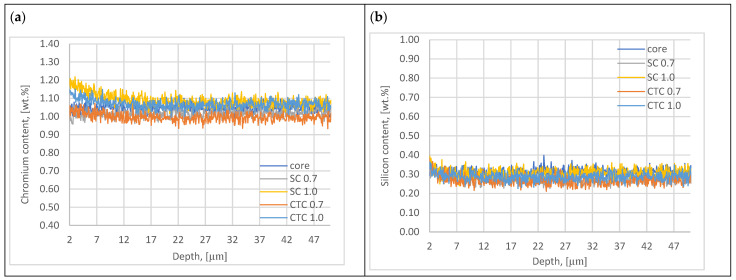
Chromium (**a**) and silicon (**b**) content plots of surface layer.

In the next step, alloying elements such as Cr and Si present in the 16MnCr5 alloy were analyzed. The results are presented in Figure 4.

Analysis of the results of chromium and silicon concentration distribution in the surface layer after low-pressure carburizing at a temperature of 1000 °C indicates that in the case of Cr, we do not observe the effect of this element escaping from the material through evaporation, as is the case with manganese. This is due to the much lower vapor pressure of chromium compared to the vapor pressure of manganese. Analyzing the data developed by Honig [15], it can be stated that for the thermodynamic conditions of carburizing presented in the work (temperature, pressure), evaporation of this element does not occur. For a temperature of 1000 °C, for the phenomenon of chromium evaporation to occur, it would be necessary to reduce the pressure in diffusion segments to a value of 0.08 Pa. In the case of chromium, however, the effect of Cr concentration increasing from the surface to a depth of ~15 µm to a value of approximately 1.2 wt.% can be observed, especially for processes carried out with the assumption of an effective layer thickness of 1 mm. This effect is less noticeable for processes designed with a layer thickness of 0.7 mm. The explanation for this may be the fact that at a temperature of 1000 °C, conditions favorable for chromium diffusion in the austenite lattice already occur [30] in the case presented in this work, additionally supported by the phenomenon of manganese escape, which causes the formation of vacancies in the crystal lattice and the tensile nature of stresses [31]. A longer exposure time at 1000 °C for 1 mm processes caused the phenomenon of core diffusion to be significant. Analyzing the graph in Figure 4a, it can be noticed that for the SC1.0 process, the increase in chromium concentration is the highest, and the explanation for this is the specificity of conducting the step carburizing process. At a temperature of 1000 °C, unlike the process carried out traditionally at a constant temperature, we already have preliminary carbon saturation in the near-surface layer. Since chromium shows a high affinity for carbon at a level of −0.084 eV [12,24,32], its diffusion to positions vacated by manganese atoms is privileged.

In the case of the second element, which is silicon, no phenomenon of this element escaping was found, nor were significant changes in concentration in the near-surface range observed, which would suggest the phenomena mentioned when describing chromium.

Also worth considering are the implications accompanying the fact of changing element concentrations in the surface layer. Such significant changes in manganese concentration in the surface layer affect the kinetics of the carburizing process itself, since, as is known, it is an element stabilizing the austenitic phase in the alloy. However, in relation to the carburizing process, a more important aspect is its interaction with carbon and direct influence on its transport into the material. The literature has shown that Mn shows significant affinity for carbon, similar to carbide-forming elements such as Cr, Mo, Ti, or W [13,33,34]. The works of Oda et al. [24] are of particular importance in the analysis of this phenomenon, where it was shown that the energy of interactions between Mn and C, which is crucial in this work, is −0.044 eV, which means that these interactions are significant and Mn has a strong affinity for C. The decrease in manganese concentration in the surface layer, therefore, affects the carbon diffusion coefficient in austenite, and thus, without taking this phenomenon into account when planning the process, the treatment effects will not be precise. The presented research results indicate (Figure 2) that both the surface concentration and the effective thickness of the carburized layer (especially in the case of processes designed for a thickness of 1 mm) present values higher than planned. The explanation for this is precisely the changes in the chemical composition of the surface layer.

### 3.2. Hardness Profile Analysis

In this chapter, it was investigated how the change in the chemical composition of the surface layer, presented in the research in Section 3.1, in addition to the influence on the kinetics of building the carburized layer, will affect the kinetics of the charge cooling process and, as a result, the final mechanical properties of the surface layer.

**Figure 5:**
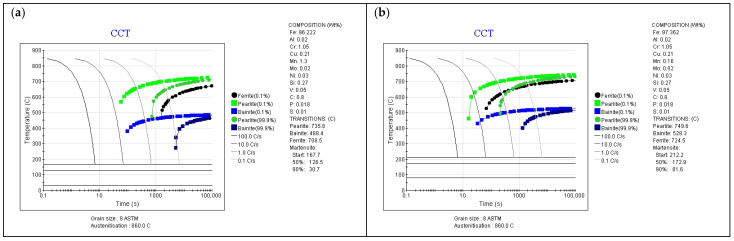

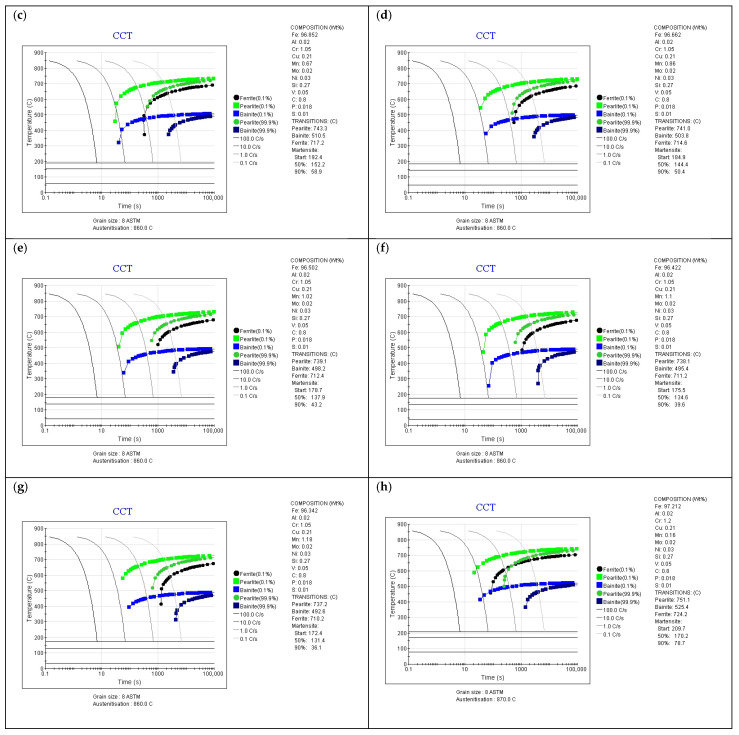
CCT diagrams for different chemical compositions of surface layer (JMatPro software), (**a**) initial composition, (**b**) composition (surface) after Mn volatilization—CTC1.0 process (**c**) composition (5 µm depth) after Mn volatilization—CTC0.7 process, (**d**) composition (5 µm depth) after Mn volatilization—SC0.7 process, (**e**) composition (10 µm depth) after Mn volatilization—CTC0.7 process (**f**) composition (10 µm depth) after Mn volatilization—SC0.7 process, (**g**) composition (15 µm depth) after Mn volatilization—SC0.7 process, (**h**) composition after Mn volatilization and Cr diffusion—CTC1.0 process.

For this purpose, the influence of these changes on the CCT diagram was first analyzed. JMatPro software version 5.1 from Sente Software Ltd. (Guildford, UK) [35] was used to determine the diagrams; the initial grain size for simulation was assumed as ASTM E112-25 [36] and the austenitizing temperature was defined as 500 °C above the calculated eutectoid transformation temperature for the adopted chemical composition. A Kirkaldy model for the calculation of ferrite and pearlite is used [37]; the following equations were developed by Zener and Hillert [38]. The Andrews model was used to calculate the start and finish temperatures of martensitic transformation [39]. The calculation results are presented in Figure 5.

Figure 5a shows the CCT diagram for the initial composition (no manganese escape from the surface layer, as well as no change in Cr concentration-hypothetical situation) after carburizing, with the assumption of carbon concentration on the surface of 0.8 wt.%C. It can be observed that to obtain a martensitic structure in the surface layer for this case, the parts should be cooled at a rate of ~9 °C/s, which, in the case of the equipment used for testing, should not be a major difficulty due to the fact that the average cooling rate for it is 20–25 °C/s.

However, the situation changes significantly if we take into account the fact of manganese escape from the material’s surface layer. For such a chemical composition of the surface layer (0.16 wt.%Mn for 1.0 processes), it was shown that the critical cooling rate required to obtain a martensite structure without the participation of equilibrium phases or bainite is ~55–60 °C/s (Figure 5b), which is beyond the capabilities of the equipment. This fact indicates that in the surface layer of the material, the existence of martensite, bainite, and equilibrium phases can be expected, which will translate into a reduction in mechanical properties in the near-surface layer. Analysis of changes in manganese concentration in the surface layer indicates that at a depth of 5 µm from the surface for the CTC0.7 process and a concentration of 0.67 wt.%Mn (Figure 5c), the required cooling rate is approximately 30 °C/s, which is slightly above the cooling capacity of the equipment, and for the SC0.7 process (Figure 5d), the required cooling rate is 10 °C/s for a concentration of 0.86 wt.%Mn, and for this process, ensuring the required cooling rate for the 16MnCr5 alloy and such manganese concentration is already achievable. Further analysis confirms that for higher manganese concentrations and greater depths in the layer, the influence of manganese concentration changes can be neglected (Figure 5e–g). Figure 5h shows a CCT diagram that takes into account the chemical composition corresponding to the SC1.0 process in the surface layer. The increase in chromium concentration, with a manganese content of 0.16 wt.%, resulted in the critical cooling rate being approximately 30 °C/min; so for this process, the influence of reduced manganese content was minimized by core chromium diffusion, while the temperatures of the start and end of martensitic transformation are close to the values for the composition with reduced Mn content.

The presented change in manganese concentration in the surface layer will have significance for the nature of martensitic transformation. As can be seen in the diagrams presented above, for initial manganese contents of 1.35 wt.%Mn and carbon concentration on the surface of 0.8 wt.%C, relative to the initial alloy, the temperatures of the start and end of martensitic transformation are significantly reduced. They are 167 °C and 30 °C, respectively. The capabilities of equipment equipped with high-pressure gas cooling are generally limited by the temperature of the cooling gas, which changes during charge cooling and is generally not lower than 35 °C. This means that for an alloy without changes in composition in the surface layer, martensitic transformation will not occur completely, and this affects the retained austenite content in the layer [40]; for such materials, charge cryogenic treatment is therefore applied. However, the phenomenon of manganese escape from the material significantly changes the temperatures of the start and end of martensitic transformation, and for the same carbon concentration in the surface layer, these values are now 212 °C and 80 °C, respectively. This means that the influence of manganese concentration changes in the surface layer may also have beneficial aspects; however, confirmation of these theses will be presented in subsequent chapters, as the reduction in hardenability may be decisive here.

**Figure 6:**
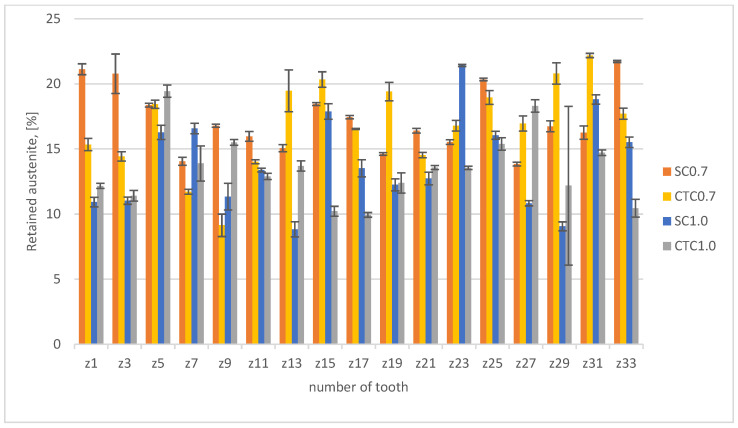
Retained austenite content.

**Figure 7:**
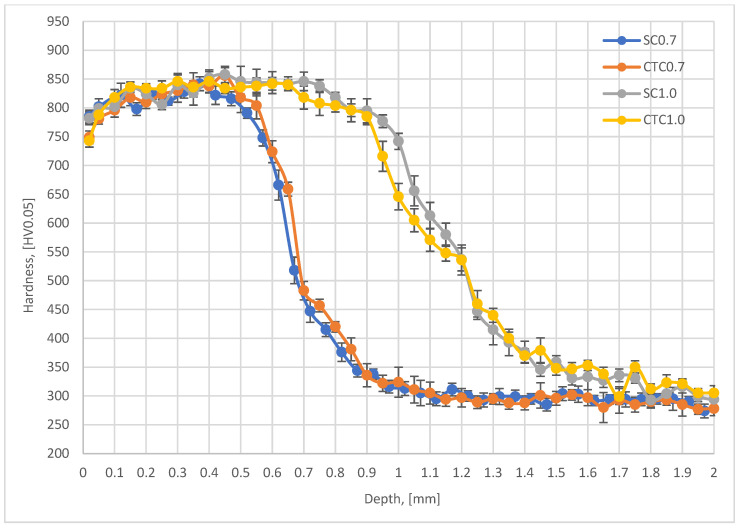
Hardness profiles for SC I CTC processes.

In the next stage, the above-mentioned factors were considered by analyzing hardness profiles and retained austenite content for individual processes. The results are presented in Figure 6 (austenite) and Figure 7 (hardness profiles).

From the analysis of the hardness graph, it follows that all processes, in the layer depth range from 0.1 to 0.4 mm, show similar average hardness at the level of 830 HV0.05. This is a typical value for this material after carburizing and quenching with stress-relief tempering. Both 0.7 mm processes and 1.0 mm processes show the convergence of profiles in this depth range, which confirms the correctness of the assumptions presented in Section 2. The effective thickness of the hardened layer for 0.7 mm processes is 0.7 mm for the hardness criterion of 500 HV. In the case of 1.0 mm processes, the effective layer thickness is 1.2 mm. It should also be noted that in all analyzed profiles, it can be seen that in the range from the surface to a depth of approximately 0.4 mm, the hardness profile slightly increases. For a depth of 0.1 mm, the average hardness value is 815 HV0.05, while for a depth of 0.4 mm, it is 840 HV0.05. This difference results from the superposition of three phenomena: retained austenite content in the surface layer, cooling rate at a given distance from the surface, and chemical composition. For a depth of 0.1 mm, we have in the material composition 1.35Mn and approximately 0.82 wt.%C, which favors a greater amount of retained austenite compared to a depth of 0.4 mm, where the Mn content is the same, but the carbon concentration is only 0.7 wt.%C for 0.7 processes and 0.75 wt.%C for 1.0 processes. This change affects the Ms and Mf temperatures, which finally allows higher hardness to be obtained. We cannot forget here about the change in Mn concentration at the surface, which, as shown earlier, significantly affects the hardenability of the material as well as the Ms and Mf temperatures. Analyzing the data for the depth where Mn change occurs, it can be noticed that the material hardness is significantly reduced there and is ~785 HV0.05 for SC0.7 and SC1.0 processes and approximately 750 HV0.05 for CTC processes. This means that at this depth, the change in chemical composition related to Mn escape and chromium concentration increase has decisive significance for mechanical properties, but, analyzing the retained austenite content in the surface layer (Figure 6), it can be noted that it is not decisive. Average austenite values in the surface layer are 16.5% for the SC0.7 process and 18% for the CTC0.7 process; for 1.0 processes designed for a layer thickness of 1 mm, the austenite content is on average 14% for both variants. There is no clear indication here of a specific solution to the method of conducting the carburizing process; however, it can be seen that in individual teeth of the gear wheel for the SC process, values are higher than for the CTC process. It seems that the change in manganese and chromium concentration has greater significance in the context of changes in critical cooling rate than in the aspect of temperatures of the start and end of martensitic transformation, especially since, for example, for teeth 13, 19, or 27, this tendency is reversed. This can be explained precisely by different heat removal intensities.

The above considerations should affect the stress state in the surface layer, and these, in turn, affect the deformation state.

**Figure 8:**
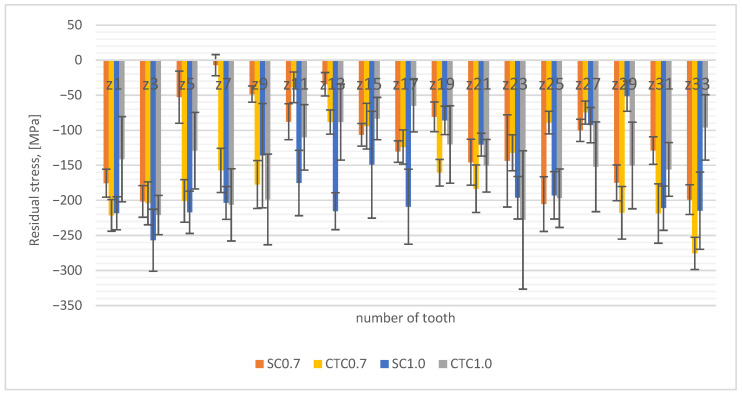
Residual stress distribution.

Therefore, XRD stress studies were carried out, and the results are presented in Figure 8.

The test results indicate that for the SC0.7 process, the measured compressive stresses are lower than in the case of the process carried out at a constant carburizing temperature and show greater differences in manganese concentrations. This can be explained similarly to the interpretation of austenite test results; in the SC process, due to smaller changes in manganese concentration, hardenability in the surface layer range is higher and results in more martensite. This also indicates that for this process, hardness is highest in the near-surface layer range (Figure 7). On the other hand, the opposite tendency can be seen for 1.0 processes, where the SC process is characterized by greater compressive stresses than the CTC process. In processes 1.0, there is another variable related to elemental composition, as was shown in Section 3.1 (Figure 4a), that for these processes, the chromium concentration increases in the near-surface layer. Analyzing the graph in Figure 4a, it can be noticed that for the SC1.0 process, the increase in chromium concentration is the highest, and the explanation for this is the specificity of conducting the step carburizing process. At a temperature of 1000 °C, unlike the process carried out traditionally at a constant temperature, we already have preliminary carbon saturation in the near-surface layer from stages carried out at lower temperatures. Since chromium shows high affinity for carbon at a level of −0.084 eV [12,24,32], its diffusion to positions vacated by manganese atoms is privileged.

### 3.3. Dimensional Change Results

**Figure 9:**
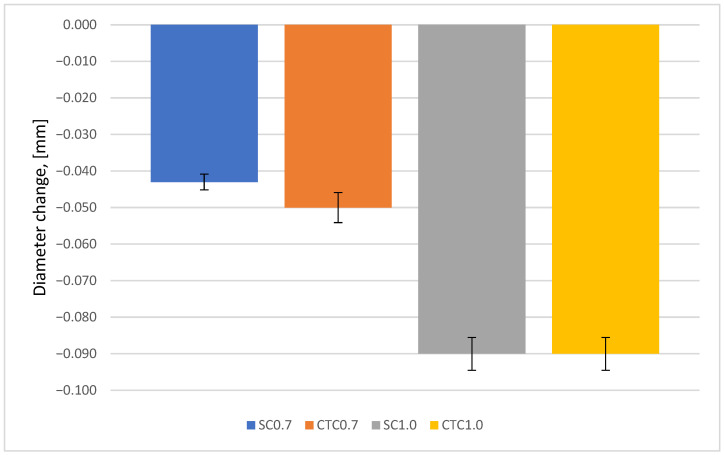
Change in mounting hole diameter of the wheel.

The analysis of dimensional changes for parts processed according to the processes presented in the research methodology section is presented below. First, changes in the diameter of the wheel mounting hole were analyzed (Figure 9).

It can be seen that for all carburizing processes, regardless of their implementation method, a decrease in the hole dimension occurred, measured at half its height. For 0.7 processes, these changes are on average 50% smaller than for 1.0 processes, which results directly from the thickness of the carburized layer. The depth of the hardened layer for 1.0 processes is significantly greater, and this directly translates into the magnitude of deformations. No differences were observed due to whether the process was carried out at constant temperature or step carburizing was used. In the case of 0.7 processes, a difference in dimensional changes is revealed here and amounts to ~7 µm.

**Figure 10:**
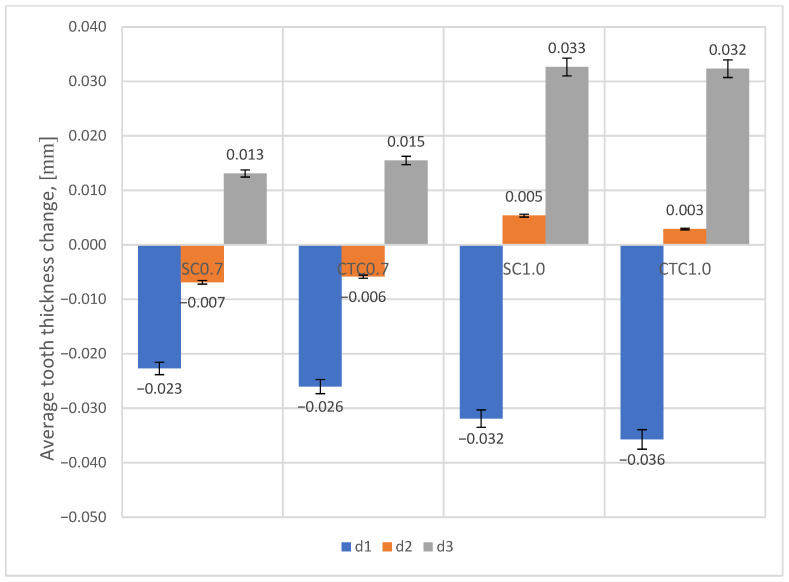
Change in tooth thickness for individual processes at tip diameter (d1), pitch diameter (d2), and root diameter (d3).

Similar conclusions can be drawn by analyzing changes in average tooth thickness for individual processes (Figure 10).

With the increasing thickness of the hardened layer after the carburizing process, the deformation value increases. On the tip diameter (d1), it can be noticed that a decrease in dimension occurs, similar to the case of the mounting hole diameter. This can be attributed to the fact that for this diameter we have higher cooling rates than for the pitch diameter (d2) or root diameter (d3), which in turn causes the appearance of martensite structure and compressive stresses. For the pitch diameter (d2), depending on the type of carburizing process carried out, deformations in the surface layer change from negative for 0.7 processes (dimension reduction) to positive for 1.0 processes (dimension increase). For the root diameter (d3), in all presented cases, we are dealing with an increase in dimension. This tendency results from different cooling rates across the layer cross-section in the tested areas, and consequently, different phase structures.

Analyzing individual process pairs (0.7 and 1.0), it can be noticed that smaller dimensional changes occur for SC processes, although the magnitude of these differences is not large.

**Figure 11:**
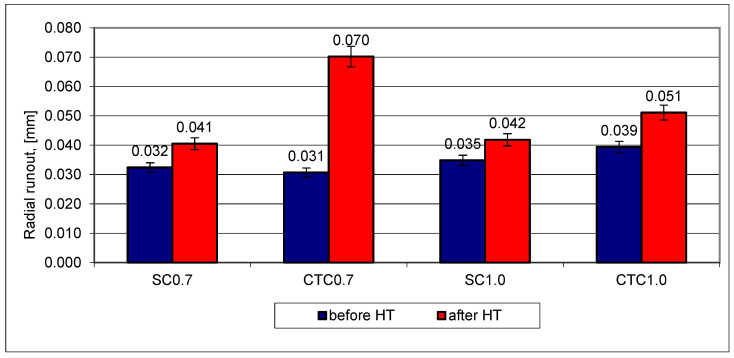
Outer radial runout of gear wheels for designed processes.

Much larger differences can be noticed when examining the outer radial runout on the tested gears (Figure 11).

In the case of step carburizing processes, regardless of the hardened layer thickness, dimensional changes are very similar and do not exceed 10 µm. Slightly larger deformations for outer radial runout can be noticed for the CTC1.0 process, where average deformation values are 12 µm. However, significant differences were shown for the CTC0.7 process, where the average radial runout change value is ~40 µm. In the case of the analysis of this geometric quantity, the influence of the carburizing method for layers not exceeding 0.7 mm is clearly evident. Preliminary saturation of the surface layer with carbon at lower temperatures and smaller changes in the concentration of alloying elements in the 16MnCr5 alloy significantly limited dimensional changes for radial runout. For thicker carburized layers, this tendency does not occur or is negligible.

## 4. Conclusions

The research conducted proves the phenomenon of manganese escape from the surface layer of parts subjected to low-pressure carburizing treatment. The following has been demonstrated:

∘ This phenomenon increases with holding time at carburizing temperature, and the intensity of this phenomenon can be limited by using step carburizing.
∘ Changes in manganese concentration affect the critical cooling rate value and Ms and Mf temperatures, which in turn translate into retained austenite content, stresses, and hardness across the layer section.
∘ Changes in chemical composition and their implications in phase transformation and stress studies constitute justification for the occurring geometric changes. The SC0.7 process shows the smallest geometric changes, where the smallest changes in chemical composition after the process were also observed. In relation to the process carried out at constant temperature, these changes are not very large, except for deformations concerning outer radial runout.
∘ The influence of manganese concentration changes is most visible in the analysis of dimensional changes for tooth thickness and mounting hole diameter. Here, these changes can be seen to follow changes in manganese concentration.

## Figures and Tables

**Table 1:** Chemical composition of material used.

	C	Mn	Si	P	S	Cr	Ni	Mo	V	Cu	Al	Fe
wt.%	0.16	1.3	0.23	0.019	0.009	1.05	0.1	0.02	0.00	0.2	0.028	96.88

**Table 2:** Details of process time.

	SC0.7	CTC0.7	SC1.0	CTC1.0
Carb. time 850 °C [min]	2	0	2	0
Carb. time 900 °C [min]	1	0	1	0
Carb. time 950 °C [min]	1	0	1	0
Carb. time 1000 °C [min]	7	9.5	16	16
Diff. time 1000 °C [min]	40	54	137	151

## Data Availability

The original contributions presented in this study are included in the article. Further inquiries can be directed to the corresponding author.

## Abbreviations

The following abbreviations are used in this manuscript:

SC	step carburizing
CTC	constant time carburizing
GDOES	glow discharge optical emission spectroscopy
QDP	quantitative depth profiling
XRD	X-ray diffraction
CCT	continuous cooling transformation
HV	Vickers hardness
Carb. Time	carburizing time
Diff. time	diffusion time
